# Brevibacterium casei: An Unusual Cause of Peritoneal Dialysis-Associated Peritonitis Managed by Catheter Salvage

**DOI:** 10.7759/cureus.114319

**Published:** 2026-08-10

**Authors:** Bruno Pepe, Maria Inês Roxo, Gonçalo Pimenta, Ana Rita Calça, Patricia Matias, Patrícia Branco

**Affiliations:** 1 Department of Nephrology, Hospital de Santa Cruz, Lisbon, PRT; 2 Navy Health Directorate, Marinha Portuguesa, Lisbon, PRT

**Keywords:** automated peritoneal dialysis, brevibacterium casei, catheter salvage, chronic kidney disease, intraperitoneal antibiotics, peritoneal dialysis, peritonitis, rare pathogens

## Abstract

Peritoneal dialysis (PD)-associated peritonitis remains the leading infectious complication of the technique and a major cause of catheter loss and transfer to haemodialysis. Although it is usually caused by common Gram-positive organisms, rare pathogens such as *Brevibacterium casei* are occasionally implicated. These coryneform bacteria are easily mistaken for skin contaminants, since they are part of the commensal flora, and may require molecular methods for identification. They are typically resistant not only to first-line beta-lactams but also to third-generation cephalosporins, clindamycin and trimethoprim-sulfamethoxazole, while usually remaining susceptible to vancomycin.

We report a rare case of *B. casei *PD-associated peritonitis in a 38-year-old man on automated PD awaiting simultaneous kidney-pancreas transplantation. Prompt microbiological identification enabled targeted intraperitoneal antibiotic therapy, resulting in complete clinical and cytological resolution, catheter preservation, and no relapse.

This case highlights the potential for catheter salvage in selected cases, with early diagnosis, organism-directed treatment, and close monitoring.

## Introduction

Peritoneal dialysis (PD) is a widely used kidney replacement therapy that supports patient autonomy and helps preserve vascular access and residual kidney function. However, peritonitis remains the leading infectious complication of the technique and a major cause of catheter loss, transfer to haemodialysis and mortality. Catheter preservation is therefore, whenever possible and safe, essential to maintain the advantages that make PD valuable. Population-based registry data indicate that, although peritonitis rates have declined in some centers, they remain substantial worldwide [1]. In the international PD Outcomes and Practice Patterns Study (PDOPPS), Gram-positive organisms accounted for the largest proportion of episodes, and peritonitis was strongly associated with adverse outcomes, including death [2]. The growing recognition of unusual causative organisms, aided by matrix-assisted laser desorption/ionisation time-of-flight (MALDI-TOF) mass spectrometry and 16S ribosomal RNA (rRNA) gene sequencing, has meaningful diagnostic and therapeutic implications [3].

*Brevibacterium casei* is a coryneform, Gram-positive rod that is part of the normal human skin microbiota and is also found in dairy products and environmental sources. Once dismissed as a culture contaminant, it is now recognised as an opportunistic pathogen, particularly in patients with malignancy, indwelling vascular catheters, immunosuppression, or end-stage kidney disease (ESKD) on PD [3,4]. In the largest review of human *Brevibacterium* infections to date, bacteraemia was the most frequent presentation, and PD-associated infection the second, with *B. casei* the most commonly identified species. Reported syndromes also include meningitis, endocarditis and device-related sepsis, with an overall mortality of approximately 10% [3]. Most isolates remain susceptible to vancomycin but show high rates of resistance to beta-lactams, trimethoprim-sulfamethoxazole and clindamycin; therefore, vancomycin is favoured for empirical treatment while susceptibility results are awaited [3,4].

Even so, *B. casei* peritonitis is exceedingly rare, and the few published cases have often been characterised by delayed identification, poor response to standard empirical regimens and catheter loss [5-7]. Accordingly, the 2022 International Society for Peritoneal Dialysis (ISPD) guideline emphasises prompt evaluation, inoculation of effluent into blood-culture bottles to maximise organism recovery, susceptibility-guided therapy and structured assessment of treatment failure [8]. Herein, we present a case of *B. casei* peritonitis in a young man on automated PD (APD) awaiting simultaneous kidney-pancreas transplantation, in whom early organism-directed therapy achieved cure with catheter salvage, an outcome reported in only a minority of previous cases [9,10].

## Case presentation

A 38-year-old Caucasian man with ESKD undergoing APD presented with a 24-hour history of diffuse abdominal pain and cloudy peritoneal effluent. The pain was moderate and worsened during fluid exchanges. He reported no fever, nausea, vomiting, diarrhoea, or constipation. His past medical history included type 1 diabetes mellitus complicated by diabetic nephropathy, retinopathy, and neuropathy, as well as hypertension and glaucoma. Kidney replacement therapy had been initiated as continuous ambulatory PD the previous year and was subsequently transitioned to APD. He had experienced one previous peritonitis episode three months prior to the reported episode, caused by oxacillin-sensitive *Streptococcus mitis*, which was successfully treated with a three-week course of intraperitoneal cefazolin. He maintained good functional capacity, remained professionally active, and was on the active waiting list for simultaneous kidney-pancreas transplantation. 

His APD regimen comprised four nightly exchanges of 2,000 mL each: two 1.36% dextrose solutions, one 1.1% amino-acid solution, and one 7.5% icodextrin solution. A previous peritoneal equilibration test showed a high peritoneal transporter status (dialysate-to-plasma creatinine ratio of 0.82), peritoneal ultrafiltration of 400-700 mL, and total fluid removal of approximately 1,020 mL/24 h, with a weekly Kt/V of 1.76.

Over the preceding 24 hours, diuresis was 300 mL and net ultrafiltration was 920 mL. Intermittent fluid overload had been documented over the preceding weeks. Physical examination was unremarkable. Laboratory and peritoneal effluent findings at presentation, together with the corresponding reference ranges, are summarised in Table 1. The peritoneal effluent met the diagnostic criterion for PD-associated peritonitis of more than 100 cells/µL with more than 50% neutrophils [11].

**Table 1:** Laboratory and peritoneal effluent findings at presentation Reference ranges are those of the local clinical laboratory for adult patients. Peritoneal effluent leucocyte and neutrophil thresholds correspond to the diagnostic criteria for PD-associated peritonitis (> 100 cells/µL with > 50% neutrophils) [11]; no formal reference range is established for effluent erythrocytes, and the value shown reflects the upper limit of what is usually observed in uncomplicated PD effluent. PD, peritoneal dialysis

Parameter	Patient value	Reference range
Blood		
Haemoglobin (g/dL)	10.8	13.0–17.0
Leucocytes (× 10⁹/L)	4.2	4.0–10.0
Platelets (× 10⁹/L)	280	150–400
Glucose (mg/dL)	207	70–110
Glycated haemoglobin (%)	9.4	4.0–5.6
Urea (mg/dL)	127	13–43
Creatinine (mg/dL)	5.88	0.70–1.20
Albumin (g/dL)	3.7	3.5–5.2
Sodium (mmol/L)	133	136–145
C-reactive protein (mg/dL)	0.15	< 0.50
Peritoneal effluent		
Leucocytes (cells/µL)	3,152	< 100
Neutrophils (%)	66	< 50
Erythrocytes (cells/µL)	1,065	< 50

Empirical intraperitoneal cefazolin (1.5 g) and ceftazidime (1.5 g) were started according to the local protocol, providing Gram-positive and Gram-negative coverage, respectively, in line with ISPD recommendations [8].

After 72 hours, *B. casei* was isolated from peritoneal effluent culture and confirmed by MALDI-TOF. As Brevibacterium spp. are characteristically resistant to beta-lactams and cephalosporins, therapy was switched to intraperitoneal vancomycin, administered during the overnight dwell for three weeks; with therapeutic drug monitoring guiding treatment, serum vancomycin concentrations were measured periodically to guide dose adjustment and target a trough level above the ISPD-recommended threshold [8]. Antimicrobial susceptibility profile revealed resistance to benzylpenicillin and clindamycin, and susceptibility to vancomycin and ciprofloxacin (increased exposure). 

The patient improved rapidly, with resolution of abdominal pain and a favourable overall course. Dialysate cytology normalised after treatment optimisation (peritoneal effluent containing 32 cells/µL on the last evaluation), and catheter function was preserved throughout follow-up, with no signs of relapse, allowing catheter salvage.

## Discussion

Peritonitis is the most common serious complication of PD and a leading cause of technique failure. Although Gram-positive cocci predominate, uncommon pathogens pose particular challenges for diagnosis and treatment because they may not be adequately covered by standard empirical regimens and may resist routine identification [2,8].

Coryneform organisms, such as *Brevibacterium spp.*, are often dismissed as skin contaminants and may require prolonged incubation or advanced methods (such as MALDI-TOF mass spectrometry or 16S rRNA gene sequencing) for definitive identification, which can delay targeted therapy [3,5]. Inoculating dialysis effluent directly into blood-culture bottles, as recommended by the ISPD, improves organism recovery and shortens the time to identification [8]. When coryneform organisms are isolated, confirmation by advanced methods is advisable to avoid misidentification and, through clinical correlation, to distinguish true infection from contamination [3].

Because *Brevibacterium spp*. are typically resistant to beta-lactams used in first-line empirical PD regimens but susceptible to vancomycin, prompt adjustment to a glycopeptide is pivotal once a coryneform organism is identified [3,9]. ISPD guidelines recommend two weeks of therapy for most Gram-positive peritonitis; however, given this organism's propensity for relapse, we elected to treat for three weeks, consistent with previously reported strategies [8,9].

Outcomes in *B. casei* peritonitis have been variable (Table 2): catheter removal and transfer to haemodialysis were required in several patients, whereas catheter salvage with vancomycin has been achieved in only a minority [5-7,9,10]. Our patient's rapid response and successful catheter salvage are therefore noteworthy and align with the favourable courses reported by Roy et al. and Wijtvliet et al. [9,10]. Catheter preservation was particularly valuable here, as maintaining PD in a kidney-pancreas transplant candidate avoids the vascular-access and allosensitisation concerns associated with haemodialysis.

**Table 2:** Reported cases of Brevibacterium casei peritoneal dialysis-associated peritonitis, with the present case CAPD, continuous ambulatory peritoneal dialysis; APD, automated peritoneal dialysis; IP, intraperitoneal; PD, peritoneal dialysis; F, female; M, male.

Study (year)	Country	Age/sex	PD modality	Treatment	Outcome
Poesen et al. (2012) [5]	Belgium	37/M	CAPD	IP vancomycin (recurrent relapses)	Catheter removal required for eradication
Althaf et al. (2014) [6]	Saudi Arabia	33/F	APD	IP cefazolin + ceftazidime (two weeks)	Catheter removal; switch to haemodialysis
Roger et al. (2024) [7]	France	75/M	CAPD	IP vancomycin (three weeks) after relapse; oral clindamycin (2 weeks) after catheter removal	Catheter removal after second relapse; later resumption of PD
Roy et al. (2022) [9]	USA	63/M	APD	IP vancomycin (three weeks)	Catheter salvage
Wijtvliet et al. (2025) [10]	Belgium	62/M	APD	IP vancomycin (three weeks)	Catheter salvage
Our case report	Portugal	38/M	APD	IP vancomycin (three weeks)	Catheter salvage

This case report describes a single case, limiting generalisability, and species identification relied on culture-based methods; nonetheless, the clinical course, susceptibility profile, and treatment response were internally consistent and concordant with the published literature [3,10]. The case reinforces that early recognition of an atypical organism, guideline-anchored management, and timely organism-directed therapy can together preserve the catheter.

## Conclusions

*B. casei *is a rare cause of PD-associated peritonitis that should be considered when cultures reveal coryneform organisms or when standard empirical therapy fails to yield the expected clinical improvement. Early diagnosis, susceptibility-guided treatment, and adherence to ISPD management principles may enable cure with catheter salvage, as demonstrated in this patient, in whom catheter preservation also helped preserve future transplant options. Given the scarcity of reported cases and the variability in outcomes, continued publication of cases of *B. casei *peritonitis is important to improve recognition, refine management, and support evidence-based decision-making in PD programmes.
